# Functional and Mortality Outcomes with Medical and Surgical Therapy in Malignant Posterior Circulation Infarcts: A Systematic Review

**DOI:** 10.3390/jcm12093185

**Published:** 2023-04-28

**Authors:** Nicole-Ann Lim, Hong-Yi Lin, Choon Han Tan, Andrew F. W. Ho, Tseng Tsai Yeo, Vincent Diong Weng Nga, Benjamin Y. Q. Tan, Mervyn J. R. Lim, Leonard L. L. Yeo

**Affiliations:** 1 Department of Medicine, Yong Loo Lin School of Medicine, National University of Singapore, Singapore 117597, Singapore; 2 Lee Kong Chian School of Medicine, Nanyang Technological University, Singapore 636921, Singapore; 3 Department of Emergency Medicine, Singapore General Hospital, Singapore 169608, Singapore; 4 Pre-Hospital & Emergency Care Research Centre, Duke-NUS Medical School, Singapore 169547, Singapore; 5 Division of Neurosurgery, Department of Surgery, National University Health System, Singapore 119074, Singapore; 6 Division of Neurology, Department of Medicine, National University Health System, Singapore 119074, Singapore

**Keywords:** stroke, posterior circulation, neurosurgery, medical therapy

## Abstract

Background: There remains uncertainty regarding optimal definitive management for malignant posterior circulation infarcts (MPCI). While guidelines recommend neurosurgery for malignant cerebellar infarcts that are refractory to medical therapy, concerns exist about the functional outcome and quality of life after decompressive surgery. Objective: This study aims to evaluate the outcomes of surgical intervention compared to medical therapy in MPCI. Methods: In this systematic review, MEDLINE, Embase and Cochrane databases were searched from inception until 2 April 2021. Studies were included if they involved posterior circulation strokes treated with neurosurgical intervention and reported mortality and functional outcome data. Data were collected according to PRISMA guidelines. Results: The search yielded 6677 studies, of which 31 studies (comprising 723 patients) were included for analysis. From the included studies, we found that surgical therapy led to significant differences in mortality and functional outcomes in patients with severe disease. Neurological decline and radiological criteria were often used to decide the timing for surgical intervention, as there is currently limited evidence for preventative neurosurgery. There is also limited evidence for the superiority of one surgical modality over another. Conclusion: For patients with MPCI who are clinically stable at the time of presentation, in terms of mortality and functional outcome, surgical therapy appears to be equivocal to medical therapy. Reliable evidence is lacking, and further prospective studies are rendered.

## 1. Introduction

Stroke has become increasingly prevalent, with the mean global lifetime risk of stroke increasing from 22.8% in 1990 to 24.9% in 2016 [1]. Ischemic strokes account for approximately 80% of all strokes, 20% of which are posterior circulation strokes that involve the vertebral arteries, basilar artery, posterior cerebral arteries and their branches [2,3].

Posterior circulation strokes tend to have a worse prognosis than their anterior circulation counterparts, and this is partly due to the important structures located there and partly due to the difficulty in diagnosis that results in longer onset-to-door time [4]. The presentation is oftentimes non-specific, with dizziness, vertigo and vomiting as the only symptoms [5]. In addition, as compared to the anterior cranial fossa, the smaller confines of the posterior fossa rapidly lead to mass effect, brainstem compression and increased mortality.

In extensive posterior circulation infarcts, mass effect with brainstem and fourth ventricle compression, hydrocephalus and brainstem herniation can occur [3]. Medical management for this includes osmotic therapy and other ancillary measures, such as elevating the head of the bed, hypothermia, barbiturates and corticosteroids [5]. However, these are typically temporising measures until the resolution of the mass effect occurs or there is definitive decompressive surgical treatment [5]. Neurosurgical therapy for MPCI includes extraventricular drainage (EVD), suboccipital decompressive craniectomy (SDC), SDC with necrosectomy and SDC with EVD.

There is evidence for early decompressive surgery in anterior circulation malignant middle cerebral artery infarcts [6,7]; however, evidence in MPCI is limited and warrants further review. While the American Heart Association/American Stroke Association guidelines recommend craniectomy in those with MPCI that are refractory to medical therapy [5], the evidence for this is sparse [8], as there are no randomized controlled trials on posterior circulation strokes and existing meta-analysis on this topic does not include the latest published data [9,10,11]. To date, effective and sustaining conservative treatments for malignant posterior infarcts are widely in practice. Surgery is currently the mainstay for the rapid decompression of the posterior fossa such that any viable brain cells can be preserved timely, especially for patients with MPCI who are unstable. However, there is another group of MPCI patients who are relatively more stable but with the risk of deterioration that can be managed conservatively.

This paper aims to provide a narrative review of the surgical interventions against medical therapy for the treatment of MPCI in patients who are relatively stable and to investigate the optimal type and timing of neurosurgical interventions for MPCI.

## 2. Methods

The conduct and reporting of this study adhere to the Preferred Reporting Items for Systematic Reviews and Meta-Analyses (PRISMA) guidelines [12]. The study protocol has been published in the International Prospective Register of Systematic Reviews (PROSPERO, CRD42021247737).

### 2.1. Search Strategy

The following databases, MEDLINE, EMBASE and the Cochrane Library, were searched from inception until 2 April 2021 using a search strategy designed in conjunction with a medical information specialist (Medical Library, National University of Singapore). The MEDLINE search used keywords synonymous with “ischemic stroke”, “cerebellar infarction”, “posterior cerebral infarction”, “vertebral infarction”, “basilar infarction”, “occipital infarction”, “cerebral infarction”, “craniotomy”, “craniectomy”, “surgical decompression”, “ventriculostomy” and “ventriculoperitoneal shunt”. The detailed search strategy is available in Supplementary Table S1. References of included studies and grey literature sources, such as Google Scholar, were also hand-searched.

### 2.2. Inclusion and Exclusion Criteria

Studies were included if they involved patients with acute ischemic stroke involving the posterior circulation who later underwent neurosurgical intervention. Neurosurgical intervention was defined as any combination of ventriculostomy, cerebral shunting, ventricular drains, craniotomy or craniectomy, with or without necrosectomy. Randomized controlled trials, observational studies and case series with sufficient death and functional outcome data were included.

The following study designs were excluded: non-English studies without an accompanying English translation, conference abstracts, review articles, pre-clinical studies, studies involving paediatric populations, studies involving participants who only suffered from haemorrhagic stroke and studies where the indication for neurosurgery was only after medical therapy had failed.

### 2.3. Study Selection

Screening was conducted through Covidence (Melbourne, VIC, Australia), an online systematic review tool recommended by Cochrane. The studies were reviewed independently by two authors (N.A. Lim and H.Y. Lin) through two rounds of screening using their titles/abstracts and full texts. Disagreements were resolved through consensus.

### 2.4. Data Extraction

The following information was independently extracted from each article: authorship, year of publication, journal, country, hospital, study design, study period and aims. The following patient demographical data were extracted: number of participants, sex and age. Data on the following comorbidities were extracted: hypertension, hyperlipidemia, atrial fibrillation and cardiac data (myocardial infarction, coronary artery disease, congestive heart failure, ischemic heart disease and coronary disease). Pre-intervention parameters were collected, namely bilateral stroke, hydrocephalus, time from symptom onset to neurosurgical intervention and Glasgow Coma Scale (GCS) at admission and pre-operatively.

Post-intervention findings such as the following were also collected: GCS, Glasgow Outcome Scale (GOS), mRS and number of deaths. Death was defined as 1 and 6 on the GOS and mRS, respectively, or extracted from the text. Deaths at all reported time points were included, which ranged from time of discharge to 57.6 months [13]. Good functional outcome was defined as mRS 0–2, GOS 4–5 and Barthel Index 91–100 or extracted from text (Table 1A,B).

### 2.5. Risk of Bias Assessment

Risk of bias of the studies were independently assessed by two authors (N.A. Lim and H.Y. Lin) using the Newcastle–Ottawa Scale [14]; the Joanna Briggs Institute (JBI) Critical Appraisal Checklist for analytical case-control study [15]; and the JBI Critical Appraisal Checklist for Case Series [16] for observational studies, case-control studies and case series, respectively.

### 2.6. Reporting Bias Assessment

The relevant authors were contacted if there was missing data that was essential for our analysis. 

**Table 1:** (**A**) General characteristics of studies of patients with posterior ischemic stroke who are treated surgically or medically. (**B**) General characteristics of studies of patients with posterior ischemic stroke who are treated surgically only.

(A)							
Study Title	Authors	Study Design	Country	Definition of Good Functional Outcome	Number of Patients Treated Surgically	Number of Patients Treated Medically	Follow-Up Duration (Months)
Cerebellar infarction with obstructive hydrocephalus	Taneda et al., 1982 [17]	Retrospective cohort study	Japan	Completely recovered	10	5	Unreported
Surgical and medical management of patients with massive cerebellar infarctions: results of the German–Austrian Cerebellar Infarction Study.	Jauss et al., 1999 [18]	Cohort study	Germany	mRS ≤ 2	48	36	Mean: 3
Space occupying cerebellar infarction	Hornig et al., 1994 [19]	Retrospective cohort study	Germany	mRS ≤ 1	36	16	Unreported
Neurosurgical management of cerebellar haematoma and infarct	Mathew et al., 1995 [20]	Retrospective cohort study	UK	GOS: unspecified by author. Assumed to be GOS ≥ 4	16	34	Unreported
Neuroimaging in deteriorating patients with cerebellar infarcts and mass effect	Koh et al., 2000 [21]	Retrospective cohort study	USA	mRS ≤ 2	9	26	Median: 16 (range: 1–105)
Management of acute cerebellar infarction: one institution’s experience	Raco et al., 2003 [22]	Retrospective case series	Italy	GOS: unspecified by author. Assumed to be GOS ≥ 4	19	25	Unreported
Neurosurgical management of massive cerebellar infarct outcome in 53 patients	Mostofi, 2013 [23]	Retrospective cohort study	French West Indies	Unreported by author. Unable to determine	25	28	Unreported
Predicting Surgical Intervention in Cerebellar Stroke: A Quantitative Retrospective Analysis	Taylor et al., 2020 [24]	Retrospective cohort study	USA	Unreported by author. Unable to determine	21	65	Unreported
**(B)**							
**Study Title**	**Authors**	**Study Design**	**Country**	**Definition of Good Functional Outcome**	**Number of Patients Treated Surgically**		**Follow-Up Duration (Months)**
Treatment of cerebellar infarction by decompressive suboccipital craniectomy	Chen et al., 1992 [25]	Case series	Germany	Barthel Index; unspecified by author. Assumed to be BI = 100	11		Mean: 42.9
Management of cerebellar infarction with associated occlusive hydrocephalus	Bertalanffy et al., 1992 [26]	Case series	Germany	Unreported	10		Unreported
Monitoring therapeutic efficacy of decompressive craniotomy in space occupying cerebellar infarcts using brain-stem auditory evoked potentials	Krieger et al., 1993 [27]	Case series	Germany	Unreported by author. Unable to determine	11		Unreported
Is decompressive craniectomy for acute cerebral infarction of any benefit?	Koh et al., 2000 [28]	Case series	Singapore	GOS ≥ 4	3		Mean: 7 (range: 3–17)
Clinical outcome following surgical treatment for bilateral cerebellar infarction.	Tsitsopoulos et al., 2011 [13]	Case series	Denmark	mRS ≤ 2	10		Median: 57.6 (range: 15–118)
Endoscopic third ventriculostomy for occlusive hydrocephalus caused by cerebellar infarction	Baldauf et al., 2006 [29]	Case series	Germany	Unreported by author. Unable to determine	10		Mean: 43
Controversy of surgical treatment for severe cerebellar infarction	Kudo et al., 2007 [30]	Case series	Germany	GOS	25		Unreported
Occlusive hydrocephalus associated with cerebellar infarction treated with endoscopic third ventriculostomy: report of 5 cases	Yoshimura, et al., 2007 [31]	Case series	USA	GOS; undefined. Assumed to be GOS ≥ 4	5		Mean: 3
Long-term outcome after suboccipital decompressive craniectomy for malignant cerebellar infarction.	Pfefferkorn T et al., 2009 [32]	Case series	Germany	mRS ≤ 3	57		Unreported
Long-term outcome after surgical treatment for space-occupying cerebellar infarction: experience in 56 patients.	Jüttler et al., 2009 [33]	Case series	Germany	mRS ≤ 2	56		Unreported
Hydrocephalus in posterior fossa lesions: ventriculostomy and permanent shunt rates by diagnosis	Mangubat et al., 2009 [34]	Case series	USA	Unreported by author. Unable to determine	4		Unreported
Endoscopic third ventriculostomy in patients with secondary triventricular hydrocephalus from a haemorrhage or ischaemia in the posterior cranial fossa	Vindigni et al., 2010 [35]	Case series	Italy	GOS; undefined. Assumed to be GOS ≥ 4	19		Mean: 6
Surgical treatment of patients with unilateral cerebellar infarcts: clinical outcome and prognostic factors.	Tsitsopoulos et al., 2011 [36]	Case series	Germany	mRS ≤ 2	32		Unreported
Ventriculosubgaleal shunt in the management of obstructive hydrocephalus caused by cerebellar infarction	Moussa et al., 2013 [37]	Case series	Germany	Unreported by author. Unable to determine	10		Mean: 6
Lesions on DWI and the Outcome in Hyperacute Posterior Circulation Stroke	Lee et al., 2014 [38]	Case series	South Korea	mRS ≤ 2	9		Mean: 3
Preventive suboccipital decompressive craniectomy for cerebellar infarction: a retrospective matched case control study	Kim et al., 2016 [39]	Case-control	South Korea	mRS ≤ 2	84		Mean: 12
Neurologic Outcome After Decompressive Craniectomy: Predictors of Outcome in Different Pathologic Conditions	Goedemans et al., 2017 [40]	Case series	Amsterdam	GOS ≥4	10		Mean: 12
Strokectomy and Extensive Cerebrospinal Fluid Drainage for the Treatment of Space-Occupying Cerebellar Ischemic Stroke	Tartara et al., 2018 [41]	Case series	Germany	mRS ≤ 2	11		Mean: 33.8 (range 12–58)
Long-term functional outcome after decompressive suboccipital craniectomyfor space-occupying cerebellar infarction	Lindeskog et al., 2019 [42]	Case series	Denmark	mRS ≤ 3	22		Mean: 12
Evaluation of clinical significance of decompressive suboccipital craniectomy on the prognosis of cerebellar infarction	Suyama et al., 2019 [43]	Case series	Japan	mRS; unspecified by author. Assumed to be Mrs ≤ 2	14		Mean: 3
Posterior Fossa Surgery for Stroke: Differences in Outcomes Between Cerebellar Hemorrhage and Infarcts	Lee et al., 2020 [10]	Case series	Germany	mRS ≤3	50		Mean: 44.5 ± 33.9
Cerebellar Necrosectomy Instead of Suboccipital Decompression: A Suitable Alternative for Patients with Space-Occupying Cerebellar Infarction	Hernández-Durán et al., 2020 [44]	Case series	Germany	GOS ≥ 4	34		Unreported
The impact of emergent suboccipital craniectomy upon outcome and prognosis of massive cerebellar infarction: A single institutional study	Mattar et al., 2021 [45]	Case series	Egypt	mRS ≤ 2	42		Mean: 3

BI, Barthel index; EVD, Extraventricular drainage; GOS, Glasgow Outcome scale; mRS, modified Rankin scale.

## 3. Results

**Figure 1:**
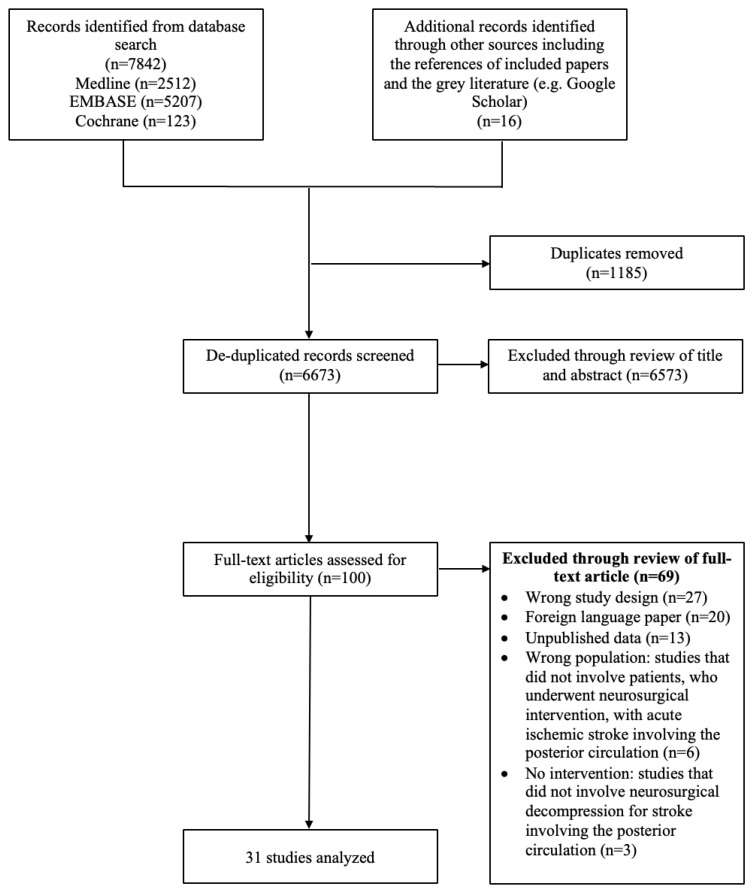
PRISMA flow diagram of included studies.

Our search yielded 6673 studies after deduplication. Following the title/abstract and full-text screen, 31 articles [10,13,17,18,19,20,21,22,23,24,25,26,27,28,29,30,31,32,33,34,35,36,37,38,39,40,41,42,43,44,45] were included for analysis. (Figure 1).

The main characteristics of the studies are summarized in Table 1A,B. Of the 31 studies included, 8 studies were observational studies that compared neurosurgery and medical therapy. The focus of this review will be on 419 patients included in these 8 dual-arm studies. Among these patients, 184 of them were treated with neurosurgery and 235 were treated with medical therapy. A total of 20 neurosurgical patients and 29 medically managed patients died. Further information containing the biodata, GCS on admission and outcome measures of the patients in the dual-arm and single-arm studies are summarized in Table 2 and Table 3, respectively. Information about the age, pre-operative GCS, comorbidities and outcome measures of all patients who underwent neurosurgery in all the studies are summarized in Table 4.

### 3.1. Medical versus Surgical Treatment

#### 3.1.1. Choice of Surgical Treatment vs. Medical Treatment

Generally, most patients receiving exclusively conservative, or medical, treatment tended to be younger [20] or have better Glasgow Coma Scale levels [20,23] than those patients for surgical intervention. However, patients presenting initially in deep comas tended to be the exception to this rule, with some institutions [20,22] opting for conservative treatment given these patients’ poor prognosis.

The treatment algorithms guiding the timing and choice of surgical treatment differed between institutions and was often left up to the discretion of individual physicians [19]. For the majority of institutions [18], the decision for surgical intervention was made based on the decline in neurological examination in conjunction with radiological criteria, such as fourth ventricular compression [13] or hydrocephalus [13,24,29]. Jauss et al. [18] found that surgery was universally performed among comatose patients, whereas treatment regimens were more diverse among patients with somnolence or stupor.

Some studies then investigated whether these clinical features used in decision making were significant predictors for surgery. Taylor et al. [24] also found that clinical features of documented brainstem compression and hydrocephalus were significant predictors. This was concordant with the findings of Koh et al. [21], who also noted an association with basal cistern compression.

#### 3.1.2. Comparing Functional Outcomes between Medical and Surgical Treatment

Studies largely agreed [18,24] that there was no significant difference in admission or discharge neurologic examination or functional status between surviving patients going through either neurosurgical or conservative management. One study by Hornig et al. [19] found that there was only a difference in functional outcome in the group of patients who were stuporous, comatose or had cardiorespiratory compromise, and surgery for this group of patients provided better functional outcomes compared to those who did not undergo surgery. This distinction between severe and limited disease was echoed by a small study by Mostofi et al. [23], which found that patients with massive ischemic cerebellar infarct, defined as ischemic volume above 5 cm^3^ and/or when there was hydrocephalus or brain stem compression, showed improvements in GCS when operated on (GCS at zero and four weeks for operated patients: 9.4 to 12.68) versus a decline when not operated on (GCS at zero and four weeks for non-operated patients: 11.36 to 10.92).

This was contradicted by a small case series of 15 patients by Taneda et al. [17], where 9 of 10 surgically operated patients survived, with the last patient dying of a perforated duodenal ulcer unrelated to the neurological insult. In that series, all five of the conservatively treated patients died.

#### 3.1.3. Comparing Mortality Rates between Medical and Surgical Treatment

**Figure 2:**
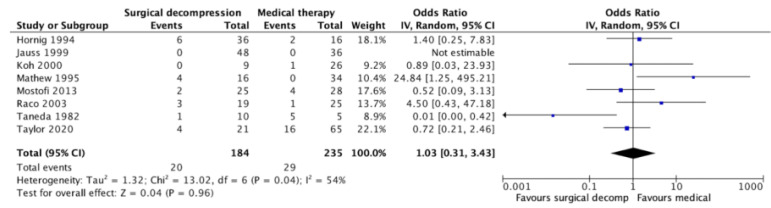
Forest plot with odds ratio (OR) and the corresponding 95% confidence interval (CI) for death in patients undergoing neurosurgical vs. medical therapy [17,18,19,20,22,23,24,28]. Events: death.

For the pooled data of 419 patients from eight studies, patients treated by neurosurgery had 3% higher odds of dying at all recorded time points as compared to patients treated by medical therapy (OR = 1.03, [95% CI: 0.31–3.43], *p* = 0.96). However, this result was not statistically significant, and there was also substantial heterogeneity among the studies (I^2^ = 54%) (Figure 2).

With neurosurgical intervention for patients with large infarcts [23] with neurologic deterioration [28] or mass effect [23,28], mortality rates dropped from 66% [23] to approximately 20% [19,23,28]. However, as noted by Jauss et al. [18], for patients who were awake or drowsy and somnolent or experiencing stupor in consciousness, there was no significant difference after 3 months in outcomes between craniotomies, ventricular drainage and medical treatment. Similar findings were reported by Hornig et al. [19] in patients with early or intermediate clinical stages as well.

### 3.2. Surgical Treatment

#### 3.2.1. Timing of Surgical Treatment

While most authors opted for surgical deterioration after clinical [18] or radiological deterioration [13,24,29], Kim et al. [39] opted for preventative craniectomies in patients with large infarcts, which was defined as a cerebellar infarction volume ratio between 0.25 and 0.33 on initial or routine follow-up radiographic findings. This was to account for patients who appeared clinically stable during the initial 72 h but would have a higher risk of delayed edema and deterioration later on. In this retrospective-matched case-control study involving 28 patients [39], preventative suboccipital decompressive craniectomy was found to have significantly better outcomes at discharge and at 12 months, and fewer deaths at 12 months.

Mattar et al. [45] also found that a short time from the onset of symptoms to surgery was significantly associated with better functional outcomes at 3 months. However, these findings were not adjusted for other variables, such as premorbid function, and this was a retrospective study without controls.

In contrast, in a retrospective study of 57 and 23 patients, respectively, Pfefferkorn et al. [32] and Lindeskog et al. [42] found that there was no significant association between time interval to surgery and outcomes.

Therefore, until there are prospective controlled studies on this topic, there remains little evidence for early or preventative craniectomies in the absence of clinical or radiological signs of deterioration.

#### 3.2.2. Choice of Surgical Intervention

Studies that were included used various combinations of EVD, SDC, SDC with necrosectomy, endoscopic third ventriculostomy (ETV), ventriculo–arterial shunts and ventriculo–peritoneal shunts. Authors [22,28] often opted for a pathophysiology-directed approach and opted for external ventricular drainage in patients with hydrocephalus. In one institution [29] with neuroendoscopic experience, endoscopic third ventriculostomy was sometimes used instead.

Juttler et al. [33] found that there was no significant difference in long-term survival and survival time in those who died between patients who were treated by SDC only, EVD only and SDC with EVD. Evidence for which treatment provided better functional outcomes was mixed, with patients treated by SDC with EVD showing better outcomes at discharge as compared to those treated by EVD alone, but long-term outcomes favouring SDC as compared to EVD alone.

When compared with pooled results from a meta-analysis [11] on SDC in cerebellar infarcts, Hernández-Durán et al. [44] found that there was no significant difference in outcomes or deaths between patients undergoing necrosectomy via osteoplastic craniotomy and patients undergoing SDC.

There is currently limited evidence for which type of neurosurgical intervention results in better outcomes. Further research should be conducted on this topic.

### 3.3. Assessment of Publication Bias

The risk of bias assessments were also assessed and summarized. Of the eight cohort studies, four were found to have poor overall quality using the Newcastle–Ottawa Scale. The remaining 24 case series and one case-control study were rated according to the Joanna Briggs Institute (JBI) Critical Appraisal Checklist (Supplementary Table S2a–c).

## 4. Discussion

Surgical therapy for malignant posterior circulation infarcts appears to have limited impact on functional outcomes and reducing mortality, except in patients with severe disease who are at a high risk of deterioration from raised intracranial pressure. Patients with posterior circulation strokes are at risk of rapid deterioration and damage to the autonomic nervous system due to the tight anatomical space in the posterior fossa and the close proximity to the brainstem. Interestingly, there are also recent studies suggesting that hypertension and diabetes are more strongly associated with posterior as compared to anterior circulation strokes. Patients with posterior circulation strokes are postulated to be more vulnerable to the atherosclerosis in metabolic diseases as the posterior circulation has finer and shorter perforating branching vessels [46,47,48]. Nonetheless, more studies are still required to explore the differences in the mechanisms and risk factors of anterior and posterior circulation strokes. Control of any existing metabolic diseases is a priority in managing patients with posterior circulation strokes.

Most authors advocate for neurosurgical intervention with the onset of symptoms, as opposed to preventative or early neurosurgical intervention. To identify severely ill patients who may benefit from neurosurgical intervention, we recommend the close monitoring of the level of arousal and for the presence of new brainstem signs, in accordance with guidelines from the American Heart Association [5]. Certain radiological criteria, such as fourth ventricular compression [13], hydrocephalus [13,24,29] or basal cistern compression [21], may also indicate a need for neurosurgical intervention.

American guidelines recommend decompressive craniectomy for patients with MPCI that have evidence of raised intracranial pressure and are imminently deteriorating. Temporizing medical therapies can also be considered. However, the overall evidence for the surgical vs. medical treatment for patients with MPCI who are still clinically stable is still weak [5]. Recent European guidelines have suggested that it should only be considered and not recommended, as there still remains uncertainty about whether such surgery improves outcomes [49]. This study aggregates preliminary evidence that surgical therapy may reduce mortality as compared to medical therapy in patients with MPCI who are clinically stable at the time of presentation, but its impact on functional outcomes is generally not significant, except in severe disease. Nonetheless, high quality trials will need to be performed to validate these findings. Moreover, there is a need to evaluate which type of neurosurgical intervention leads to better outcomes, which is beyond the scope of this study.

### Limitations

Study heterogeneity was significant, due to limited consensus on the threshold or protocol for neurosurgical treatment and different baseline characteristics of the patients. Outcome measures were variably reported, with differing times for follow-up, differing time-points when death was reported and varying definitions of good functional outcome. Further research is required to address these gaps.

There was also limited high quality data, as no large-scale randomized controlled trials were conducted on this topic. Therefore, the studies were mainly retrospective observational papers, with only one prospective study [18]. The sample sizes of the studies were also small, with the largest study involving 86 patients [24].

## 5. Conclusions

For patients with malignant posterior circulation stroke, in terms of mortality and functional outcome, surgical therapy appears to be equivocal to medical therapy. For patients with severe disease, surgery could be superior to medical therapy. There is a lack of quality data, and more randomized control trials are rendered following this review. 

## Figures and Tables

**Table 2:** Pre-intervention characteristics and post-intervention outcomes of patients with posterior circulation stroke, treated surgically or medically.

Author and Year	Raco et al., 2003 [22]						Mathew et al., 1995 [20]					
Treatment Groups	EVD Only	SDC Only	SDC with Necrosectomy Only	SDC and EVD	Others	Medical Only	EVD Only	SDC Only	SDC with Necrosectomy Only	SDC and EVD	Others	Medical Only
**Number, n (%)**	8 (18%)	4 (9.1%)	0	5 (11%)	Treatment-limiting decision: 2 (4.5%)	25 (57%)	7 (14%)	2 (4%)	0	0	Treatment-limiting decision: 3 (6%) Management change: 4 (8%)	34 (68%)
**Comorbidities**	Recent cardiac infarction: 6 Atrial flutter: 2 Endocarditis with vegetations: 2 Patent foramen ovale: 1						Unreported					
**Radiological findings**	*Presence of hydrocephalus*						*Presence of hydrocephalus*					
	8	0	0	5	0	0	Total: 19					
	*Presence of brainstem compression*						*Presence of brainstem compression*					
	Unreported						Total: 26					
**Male, n (%)**	24 (55%)						Unreported					
**Age in years ± SD (range)**	Median: 56 (9–83)						Mean: 57 (26–85)					
**GCS on admission**	GCS 3: 2 GCS 6: 2 GCS 9–12: 15 GCS 13: 15 GCS 14: 7 GCS 15: 3						Median: 14 (4–15)					
**Good functional outcome, n (%)**	8 (18%)	1 (2.3%)	-	4 (9.1%)	0	24 (55%)	6 (12%)	1 (2%)	-	-	Management change: 2 (4%)	34 (68%)
**Death, n (%)**	0	2 (4.5%)	-	1 (2.3%)	Treatment-limiting decision: 2 (4.5%)	1 (2.3%)	1 (2%)	1 (2%)	-	-	Treatment-limiting decision: 3 (6%) Management change: 2 (4%)	0
**Author and Year**	**Hornig et al., 1994** [19]						**Jauss et al., 1992** [18]					
**Treatment Groups**	**EVD Only**	**SDC Only**	**SDC with** **Necrosectomy Only**	**SDC and EVD**	**Others**	**Medical Only**	**EVD Only**	**SDC Only**	**SDC with** **Necrosectomy Only**	**SDC and EVD**	**Others**	**Medical Only**
**Number, n (%)**	2 (3.8%)	0	8 (15%)	4 (7.7%)	SDC + EVD + necrosectomy: 22 (42%)	16 (31%)	14 (17%)	30 (36%)	0	4 (4.8%)	0	36 (43%)
**Comorbidities**	Arterial hypertension: 33 Diabetes: 21 Hypercholesterolemia: 5 Unilateral/bilateral vertebral artery stenosis: 10 Unilateral/bilateral vertebral artery occlusion: 2 Nonrheumatic atrial fibrillation: 14 Myocardial infarction: 3						Unreported					
**Radiological findings**	*Presence of hydrocephalus*						*Presence of hydrocephalus*					
	Total: 42						Unreported					
	*Presence of brainstem compression*						*Presence of brainstem compression*					
	Total: 39						Unreported					
**Age in years ± SD (range)**	Mean: 61.2 ± 10.1						Mean: 54.5 ± 17.3	Mean: 57.4 ± 12	-	-	-	Mean: 61.2 ± 10.3
**GCS on admission**	Unreported						Unreported					
**Good functional outcome, n (%)**	18 (35%)					11 (21%)	10 (12%)	22 (26%)	-	-	-	30 (36%)
**Death, n (%)**	6 (12%)					2 (3.8%)	unreported					
**Author and Year**	**Mostofi, 2013** [23]						**Koh et al., 2000** [28]					
**Treatment Groups**	**EVD Only**	**SDC Only**	**SDC with Necrosectomy Only**	**SDC and EVD**	**Others**	**Medical Only**	**EVD Only**	**SDC Only**	**SDC with** **Necrosectomy Only**	**SDC and EVD**	**Others**	**Medical Only**
**Number, n (%)**	6 (11%)	16 (30%)	0	3 (5.7%)	0	28 (53%)	6 (17%)	2 (5.7%)	0	1 (2.9%)	0	26 (74%) (2 patients with treatment limiting decision)
**Comorbidities**	Unreported						Large artery disease: 13 Cardioembolism: 12					
**Radiological findings**	*Presence of hydrocephalus*						*Presence of hydrocephalus*					
	Unreported						Total among surgical group: 9					6
	*Presence of brainstem compression*						*Presence of brainstem compression*					
	Unreported						Total among surgical group: 7					2
**Male, n (%)**	32 (60%)						Unreported					
**Age in years ± SD (range)**	Mean: 58.7 (SD unreported)						Unreported					
**GCS on admission**	Mean: 9.5	Mean: 9.43	-	Mean: 6	-	Mean: 11.6	Unreported					
**Good functional outcome, n (%)**	unreported						2 (5.7%)	0	-	0	-	14 (40%)
**Death, n (%)**	2 (3.8%)					4 (7.5%)	0					1 (2.9%)
**Author and Year**	**Taneda et al., 1982** [17]						**Taylor et al., 2020** [24]					
**Treatment Groups**	**EVD Only**	**SDC Only**	**SDC with Necrosectomy Only**	**SDC and EVD**	**Others**	**Medical Only**	**EVD Only**	**SDC Only**	**SDC with Necrosectomy Only**	**SDC and EVD**	**Others**	**Medical Only**
**Number, n (%)**	0	10 (67%)	0	0	0	5 (20%)	2 (2.3%)	0	12 (14%)	9 (10%)	0	65 (76%)
**Comorbidities**	Unreported						Obese, BMI ≥ 30: 37 Hypertension: 63 Diabetes: 37 Coronary artery disease: 21 Congestive heart failure: 16 Prior cerebrovascular accident: 16 Chronic kidney disease: 8 Alcohol abuse: 22 Tobacco abuse: 23 Hyperlipidemia: 35					
**Radiological findings**	*Presence of hydrocephalus*						*Presence of hydrocephalus*					
	Total: 15						Total among surgical group: 11					5
	*Presence of brainstem compression*						*Presence of brainstem compression*					
	Unreported						Total among surgical group: 10					8
**Male, n (%)**	-	9 (60%)	-	-	-	4 (27%)	12 (14%)					41 (48%)
**Age in years ± SD (range)**	-	Mean: 55.1 (40–66)	-	-	-	Mean: 67.6 (41–80)	Median: 58.5 IQR: 52–65					
**GCS on admission**	-	unreported	-	-	-	unreported	Median: 14 (IQR: 10–15)					Median: 15 (IQR: 10–15)
**Good functional outcome, n (%)**	-	7 (47%)	-	-	-	0	-	-	-	-	-	-
**Death, n (%)**	-	1 (6.7%)	-	-	-	5 (20%)	4 (4.7%)					16 (19%)

ETV, Endoscopic third ventriculostomy; EVD, Extraventricular drainage; GCS, Glasgow Coma Scale; IQR, interquartile range; SD, standard deviation; SDC, suboccipital decompressive craniotomy.

**Table 3:** Post-intervention characteristics and post-intervention outcomes of patients with posterior circulation stroke, treated by surgery only.

Author and Year	Tsitsopoulos et al., 2010 [36]					Baldauf et al., 2006 [29]				
Treatment Groups	EVD Only	SDC Only	SDC with Necrosectomy Only	SDC and EVD	Others	EVD Only	SDC Only	SDC with Necrosectomy Only	SDC and EVD	Others
**Number, n (%)**	0	0	0	10 (100%)	0	0	0	0	0	ETV: 7 (70%) ETV + EVD: 2 (20%) ETV + SDC: 1 (10%)
**Male, n (%)**	-	-	-	8 (80%)	-	-	-	-	-	6 (60%)
**Age in years ± SD (range)**	-	-	-	Mean: 54.9 ± 13	-	-	-	-	-	Mean: 61.8 (SD unreported)
**GCS on admission**	-	-	-	Mean: 12.3 ± 3.1	-	-	-	-	-	Mean: 11.2 (SD unreported)
**Good functional outcome, n (%)**	-	-	-	6 (60%)	-	-	-	-	-	unreported
**Death, n (%)**	-	-	-	1 (10%)	-	-	-	-	-	0
**Author and Year**	**Koh et al., 2000** [21]					**Pfefferkorn et al., 2009** [32]				
**Treatment Groups**	**EVD Only**	**SDC Only**	**SDC with Necrosectomy Only**	**SDC and EVD**	**Others**	**EVD Only**	**SDC Only**	**SDC with Necrosectomy Only**	**SDC and EVD**	**Others**
**Number, n (%)**	0	3 (100%)	0	0	0	47 (82%)	57 (100%)	0	0	Infarct evacuation: 32/57 (56%)
**Male, n (%)**	-	1 (33%)	-	-	-	-	34	-	-	-
**Age in years ± SD (range)**	-	Mean: 53.6 (SD unreported)	-	-	-	-	Mean: 59.2 ± 12.9	-	-	-
**GCS on admission**	-	Mean: 12.3 (SD unreported)	-	-	-	-	unreported	-	-	-
**Good functional outcome, n (%)**	-	2 (66%)	-	-	-	-	27 (47%)	-	-	-
**Death, n (%)**	-	1 (33%)	-	-	-	-	16 (28%)	-	-	-
**Author and Year**	**Jüttler et al., 2009** [33]					**Lee et al., 2020** [10]				
**Treatment Groups**	**EVD Only**	**SDC Only**	**SDC with Necrosectomy Only**	**SDC and EVD**	**Others**	**EVD Only**	**SDC Only**	**SDC with Necrosectomy Only**	**SDC and EVD**	**Others**
**Number, n (%)**	9 (16%)	-	8 (14%)	39 (70%)	0	0	0	0	50 (100%)	0
**Male, n (%)**	37 (66%)	-	-	-	38 (76%)	-				
**Age in years ± SD (range)**	Median: 60 (30–76)	-	-	-	Mean: 57.3 ± 12	-				
**GCS on admission**	Median: 14.5 (3–15)	-	-	-	Unreported	-				
**Good functional outcome, n (%)**	4 (7.1%)	-	4 (7.1%)	12 (21%)	-	-	-	-	30 (60%)	-
**Death, n (%)**	2 (3.6%)	-	1 (1.8%)	9 (16%)	-	-	-	-	21 (42%)	-
**Author and Year**	**Tsitsopoulos et al., 2011** [13]					**Chen et al., 1992** [25]				
**Treatment Groups**	**EVD Only**	**SDC Only**	**SDC with Necrosectomy Only**	**SDC and EVD**	**Others**	**EVD Only**	**SDC Only**	**SDC with Necrosectomy Only**	**SDC and EVD**	**Others**
**Number, n (%)**	0	0	0	32 (100%)	0	0	0	0	2 (18%)	SDC + EVD + necrosectomy: 9 (82%)
**Male, n (%)**	-	-	-	24 (75%)	-	-	-	-	7 (64%)	
**Age in years ± SD (range)**	-	-	-	64.3 ± 9.9	-	-	-	-	Mean: 54 (36–73)	
**GCS on admission**	-	-	-	Median: 12.2 (7–15)	-	-	-	-	Mean: 12.9	
**Good functional outcome, n (%)**	-	-	-	19 (59%)	-	-	-	-	2 (18%)	
**Death, n (%)**	-	-	-	10 (31%)	-	-	-	-	0	
**Author and Year**	**Moussa et al., 2013** [37]					**Tartara et al., 2018** [41]				
**Treatment Groups**	**EVD Only**	**SDC Only**	**SDC with Necrosectomy Only**	**SDC and EVD**	**Others**	**EVD Only**	**SDC Only**	**SDC with Necrosectomy Only**	**SDC and EVD**	**Others**
**Number, n (%)**	0	5 (50%)	0	5 (50%)	0	0	2 (18%)	0	9 (82%)	0
**Male, n (%)**	7 (70%)	6 (55%)								
**Age in years ± SD (range)**	15 ≤ Age < 30 years: 6 30 ≤ Age < 45 years: 3 Age ≥ 45 years: 1					Mean: 64.7 ± 9.1				
**GCS on admission**	GCS 3–9 n = 5 GCS 10–12 n = 3 GCS 13–15 n = 2					Mean: 13.6 ± 1.1				
**Good functional outcome, n (%)**	Unreported					-	2 (18%)	-	7 (64%)	-
**Death, n (%)**	-	2 (20%)	-	0	-	-	0	-	1 (9.1%)	-
**Author and Year**	**Kudo et al., 2007** [30]					**Krieger et al., 1993** [27]				
**Treatment Groups**	**EVD Only**	**SDC Only**	**SDC with Necrosectomy Only**	**SDC and EVD**	**Others**	**EVD Only**	**SDC Only**	**SDC with Necrosectomy Only**	**SDC and EVD**	**Others**
**Number, n (%)**	3 (12%)	2 (8%)	0	3 (12%)	EVD + necrosectomy: 14 (56%) Necrosectomy only: 3 (12%)	0	0	0	11 (100%)	0
**Male, n (%)**	21 (84%)					-	-	-	8 (73%)	-
**Age in years ± SD (range)**	Mean age Group A: 72 ± 6 Group B: 61 ± 15					-	-	-	Mean: 52 (30–69)	-
**GCS on admission**	Unreported					-	-	-	Unreported	-
**Good functional outcome, n (%)**	11 (44%)					-	-	-	Unreported	-
**Death, n (%)**	3 (12%)					-	-	-	4 (36%)	-
**Author and Year**	**Suyama et al., 2019** [43]					**Lindeskog et al., 2018** [42]				
**Treatment Groups**	**EVD Only**	**SDC Only**	**SDC with Necrosectomy Only**	**SDC and EVD**	**Others**	**EVD Only**	**SDC Only**	**SDC with Necrosectomy Only**	**SDC and EVD**	**Others**
**Number, n (%)**	0	5 (36%)	0	9 (64%)	0	0	0	0	22 (100%)	0
**Male, n (%)**	12(86%)					-	-	-	16 (73%)	-
**Age in years ± SD (range)**	Mean: 65 ± 12					-	-	-	Median: 53 (IQR: 45–62)	-
**GCS on admission**	Unreported					-	-	-	Median: 8 (IQR: 5–10)	-
**Good functional outcome, n (%)**	10 (71%)					-	-	-	12 (55%)	-
**Death, n (%)**	2 (14%)					-	-	-	7 (32%)	-
**Author and Year**	**Mattar et al., 2021** [45]					**Hernández-Durán, 2020** [44]				
**Treatment Groups**	**EVD Only**	**SDC Only**	**SDC with Necrosectomy Only**	**SDC and EVD**	**Others**	**EVD Only**	**SDC Only**	**SDC with Necrosectomy Only**	**SDC and EVD**	**Others**
**Number, n (%)**	0	42 (100%)	0	0	0	0	0	0	0	Necrosectomy only: 34 (100%)
**Male, n (%)**	-	36 (86%)	-	-	-	-	-	-	-	18 (53%)
**Age in years ± SD (range)**	-	Mean: 66 ± 13	-	-	-	-	-	-	-	Median: 70 (28–84)
**GCS on admission**	-	Unreported	-	-	-	-	-	-	-	Median: 11 (3–15)
**Good functional outcome, n (%)**	-	25 (60%)	-	-	-	-	-	-	-	26 (76%)
**Death, n (%)**	-	6 (14%)	-	-	-	-	-	-	-	7 (21%)
**Author and Year**	**Goedemans et al., 2017** [40]					**Yoshimura et al., 2007** [31]				
**Treatment Groups**	**EVD Only**	**SDC Only**	**SDC with Necrosectomy Only**	**SDC and EVD**	**Others**	**EVD Only**	**SDC Only**	**SDC with Necrosectomy Only**	**SDC and EVD**	**Others**
**Number, n (%)**	0	10 (100%)	0	0	0	0	0	0	0	ETV: 5 (100%)
**Male, n (%)**	Unreported				-	-	-	-	3 (60%)	
**Age in years ± SD (range)**	Unreported				-	-	-	-	Mean: 71.8 (47–92)	
**GCS on admission**	Unreported				-	-	-	-	Mean: 12.8 (8–15)	
**Good functional outcome, n (%)**	-	-	5 (50%)	-	-	-	-	-	-	3 (60%)
**Death, n (%)**	-	-	Unreported	-	-	-	-	-	-	1 (20%)
**Author and Year**	**Lee et al., 2014** [38]					**Mangubat et al., 2009** [34]				
**Treatment Groups**	**EVD Only**	**SDC Only**	**SDC with Necrosectomy Only**	**SDC and EVD**	**Others**	**EVD Only**	**SDC Only**	**SDC with Necrosectomy Only**	**SDC and EVD**	**Others**
**Number, n (%)**	0	9 (100%)	0	0	0	4 (100%)	0	0	0	0
**Male, n (%)**	-	Unreported	-	-	-	Unreported	-	-	-	-
**Age in years ± SD (range)**	-	Unreported	-	-	-	Unreported	-	-	-	-
**GCS on admission**	-	Unreported	-	-	-	Unreported	-	-	-	-
**Good functional outcome, n (%)**	-	2 (22%)	-	-	-	Unreported	-	-	-	-
**Death, n (%)**	-	Unreported	-	-	-	4 (100%)	-	-	-	-
**Author and Year**	**Vindigni et al., 2010** [35]					**Bertalanffy et al., 1992** [26]				
**Treatment Groups**	**EVD Only**	**SDC Only**	**SDC with Necrosectomy Only**	**SDC and EVD**	**Others**	**EVD Only**	**SDC Only**	**SDC with Necrosectomy Only**	**SDC and EVD**	**Others**
**Number, n (%)**	12 (63%)	0	0	0	ETV: 7 (37%)	6 (60%)	0	0	0	Ventriculo–arterial shunt: 3 (30%) Ventriculo–peritoneal shunt: 1 (10%)
**Male, n (%)**	Unretrievable					2 (20%)	-	-	-	Ventriculo–arterial shunt: 1 (10%) Ventriculo–peritoneal shunt: 1 (10%)
**Age in years ± SD (range)**	Mean: 62.3 (52–73)	-	-	-	Mean: 50.4 (23–67)	Mean: 61.8 (SD unreported)				
**GCS on admission**	Unreported					Unreported				
**Good functional outcome, n (%)**	6 (32%)	-	-	-	3 (16%)	Unreported				
**Death, n (%)**	1 (5.3%)	-	-	-	1 (5.3%)	1 (10%)	-	-	-	Ventriculo–arterial shunt: 1 (10%) Ventriculo–peritoneal shunt: 1 (10%)
**Author and Year**	**Kim et al., 2016** [39]									
**Treatment Groups**	**EVD Only**		**SDC Only**		**SDC with Necrosectomy Only**		**SDC and EVD**		**Others**	
**Number, n (%)**	0		84 (100%)		0		0		0	
**Male, n (%)**	0		52 (62%)		-		-		-	
**Age in years ± SD (range)**	-		Mean age Preventive SDC group: 59.0 ± 11.6 Non-preventive SDC group: 59.4 ± 10.9		-		-		-	
**GCS on admission**	-		Mean GCS Preventive SDC group: 12.1 ± 4.1 Non-preventive SDC group: 12.0 ± 3.8		-		-		-	
**Good functional outcome, n (%)**	-		45 (54%)		-		-		-	
**Death, n (%)**	-		6 (7.1%)		-		-		-	

ETV, Endoscopic third ventriculostomy; EVD, Extraventricular drainage; GCS, Glasgow Coma Scale; IQR, interquartile range; SD, standard deviation; SDC, suboccipital decompressive craniotomy.

**Table 4:** Summary of characteristics of all patients who underwent neurosurgery.

Study	Number of Patients	Number of Deaths	Mean Age (Years)	Mean Pre-Operative GCS	Proportion of Good Functional Outcome (%)	Proportion of Patients with Hypertension (%)	Proportion of Patients with Diabetes Mellitus (%)	Proportion of Patients with Dyslipidemia (%)	Proportion of Patients with Atrial Fibrillation (%)	Proportion of Patients with Heart Disease * (%)	Proportion of Patients with Previous Stroke (%)	Proportion of Patients with Bilateral Stroke (%)	Proportion of Patients with Hydrocephalus (%)
Baldauf et al., 2006 [29]	10	0	61.8	11.2	NA	50	NA	NA	70	NA	NA	NA	100
Bertalanffy et al., 1992 [26]	10	3	61.8	NA	NA	NA	NA	NA	NA	NA	NA	NA	100
Chen et al., 1992 [25]	11	0	54	6.27	2	27.3	NA	NA	NA	NA	NA	27.3	NA
Goedemans et al., 2017 [40]	10	NA	NA	NA	5	NA	NA	NA	NA	NA	NA	NA	NA
Hernández-Durán et al., 2020 [44]	34	7	70	7.5	26	NA	NA	NA	NA	NA	NA	26.5	55.9
Hornig et al., 1994 [19]	36	6	NA	NA	18	NA	NA	NA	NA	NA	NA	NA	NA
Jauss et al., 1992 [18]	48	NA	56.55	NA	32	NA	NA	NA	NA	NA	NA	NA	NA
Jüttler et al., 2009 [33]	56	14	60	13	20	NA	NA	NA	NA	NA	NA	14.3	NA
Kim et al., 2016 [39]	84	6	59.27	NA	45	40.5	34.5	25	41.7	3.57	13.1	42.9	NA
Koh et al., 2000 [21]	9	0	NA	NA	2	NA	NA	NA	NA	NA	NA	NA	100
Koh et al., 2000 [28]	3	1	53.57	4	2	NA	NA	NA	NA	NA	NA	0	NA
Krieger et al., 1993 [27]	11	4	52	NA	NA	NA	NA	NA	NA	NA	NA	NA	NA
Kudo et al., 2007 [30]	25	3	63	6.4	11	NA	NA	NA	NA	NA	NA	NA	NA
Lee et al., 2014 [38]	9	NA	NA	NA	2	NA	NA	NA	NA	NA	NA	NA	NA
Lee et al., 2020 [10]	50	21	57.3	NA	30	NA	NA	NA	NA	NA	NA	48	NA
Lindeskog et al., 2019 [42]	22	7	53	8	12	18.2	4.55	13.6	9.09	4.55	NA	27.3	NA
Mangubat et al., 2009 [34]	4	NA	NA	NA	NA	NA	NA	NA	NA	NA	NA	NA	NA
Mathew et al., 1995 [20]	16	7	NA	NA	9	NA	NA	NA	NA	NA	NA	NA	NA
Mattar et al., 2021 [45]	42	6	66	NA	25	NA	NA	NA	NA	NA	NA	21.4	73.8
Mostofi et al., 2013 [23]	25	2	59.67	5.33	NA	NA	NA	NA	NA	NA	NA	NA	NA
Moussa et al., 2013 [37]	10	2	NA	NA	NA	NA	NA	NA	NA	NA	NA	NA	NA
Pfefferkorn et al., 2009 [32]	57	16	59.2	NA	27	80	32	30	NA	NA	NA	37	NA
Raco et al., 2003 [22]	19	5	NA	NA	13	NA	NA	NA	NA	NA	NA	NA	NA
Suyama et al., 2019 [43]	14	2	65	NA	10	35.7	7.14	NA	14.3	14.3	21.4	57.1	85.7
Taneda et al., 1982 [17]	10	1	55.1	NA	7	NA	NA	NA	NA	NA	NA	0	NA
Tartara et al., 2018 [41]	11	1	64.7	9.27	9	NA	NA	NA	NA	NA	NA	18.2	NA
Taylor et al., 2020 [24]	21	4	55	10	NA	71.4	52.4	47.6	NA	28.6	28.6	33.3	52.4
Tsitsopoulos et al., 2011 [13]	10	1	54.9	8.9	6	20	10	10	20	10	NA	50	70
Tsitsopoulos et al., 2011 [36]	32	10	64.3	9	19	46.9	18.8	NA	18.8	15.6	NA	25	90.6
Vindigni et al., 2010 [35]	19	2	50.4	NA	9	31.6	NA	NA	36.8	NA	NA	0	NA
Yoshimura et al., 2007 [31]	5	1	71.8	9.8	3	NA	NA	NA	20	NA	NA	20	NA

NA, Not applicable as data were unreported by study. * Heart disease included myocardial infarction, coronary artery disease, congestive heart failure, ischemic heart disease and coronary disease.

## Data Availability

Please contact Mervyn Lim at mervynlim@u.nus.edu to request for the study’s data if required.

## Supplementary Materials

The following supporting information can be downloaded at: https://www.mdpi.com/article/10.3390/jcm12093185/s1.

## Abbreviations

ETV	Endoscopic third ventriculostomy
EVD	Extraventricular drainage
GCS	Glasgow Coma Scale
GOS	Glasgow Outcome Scale
JBI	Joanna Briggs Institute
MPCI	Malignant posterior circulation infarcts
mRS	Modified Rankin Scale
PRISMA	Preferred Reporting Items for Systematic Reviews and Meta-Analyses
SDC	Suboccipital decompressive craniectomy
