# Supplementary material for: Functional and Mortality Outcomes with Medical and Surgical Therapy in Malignant Posterior Circulation Infarcts: A Systematic Review
Source: J Clin Med. 2023 Apr 28;12(9):3185. doi: 10.3390/jcm12093185 (PMC10179120; doi:10.3390/jcm12093185)
Supplement: Supplementary file 1 [file jcm-12-03185-s001.zip › jcm-2139495-supplementary.pdf]

Supplementary Table S1: Search Strategy

PubMed – 2,512 results

("Brain Infarction"[MeSH Terms] OR "Ischemic Stroke"[MeSH Terms] OR "Brain Ischemia"[MeSH Terms] OR "Infarction"[MeSH Terms] OR "Ischemia"[MeSH Terms] OR ("ischemic stroke*"[Title/Abstract] OR "ischaemic stroke*"[Title/Abstract] OR "wake up stroke*"[Title/Abstract] OR "cryptogenic stroke*"[Title/Abstract] OR "cryptogenic embolism stroke*"[Title/Abstract] OR "embolic stroke*"[Title/Abstract] OR "cardioembolic stroke*"[Title/Abstract] OR "cardio embolic stroke*"[Title/Abstract] OR "cardio embolic stroke*"[Title/Abstract] OR "thrombotic stroke*"[Title/Abstract] OR "brain infarction*"[Title/Abstract] OR "brain isch*"[Title/Abstract] OR "cerebral isch*"[Title/Abstract] OR "cerebral infarction*"[Title/Abstract] OR "cerebellar infarction*"[Title/Abstract] OR "infarct*"[Title/Abstract] OR "isch*"[Title/Abstract])) AND ("posterior cerebral artery"[MeSH Terms] OR "vertebral artery"[MeSH Terms] OR "basilar artery"[MeSH Terms] OR "blood circulation"[MeSH Terms] OR "occipital lobe"[MeSH Terms] OR "cerebellum"[MeSH Terms] OR ((("posterior cerebral"[Title/Abstract] OR "vertebral"[Title/Abstract] OR "cerebral"[Title/Abstract] OR "basilar"[Title/Abstract]) AND ("arteries"[Title/Abstract] OR "artery"[Title/Abstract])) OR "posterior"[Title/Abstract] OR "fossa"[Title/Abstract] OR ("cerebell*"[Title/Abstract] OR "occipital"[Title/Abstract]))) AND ("decompression, surgical"[MeSH Terms] OR "craniotomy"[MeSH Terms] OR "neurosurgical procedure*"[MeSH Terms] OR ((("craniotomy"[Title/Abstract] OR "craniectomy"[Title/Abstract] OR "hemicraniectomy"[Title/Abstract]) AND "decompress*"[Title/Abstract]) OR "suboccipital craniectomy"[Title/Abstract] OR "neurosurgical procedures"[Title/Abstract] OR (("ventriculoperitoneal"[Title/Abstract] OR "ventriculo-peritoneal"[Title/Abstract]) AND "shunt*"[Title/Abstract]) OR "Ventriculostomy"[Title/Abstract] OR ("surgical"[Title/Abstract] AND "decompression*"[Title/Abstract])))

Embase – 5207 results

(('brain ischemia'/exp OR 'brain infarction'/exp OR 'cerebral artery disease'/exp OR 'cerebrovascular accident'/exp OR 'occlusive cerebrovascular disease'/exp OR 'wallenberg syndrome'/exp OR 'ischemia'/exp OR 'infarction'/exp) OR ('ischemic stroke*':ab,ti OR 'wake up stroke*':ab,ti OR 'cryptogenic stroke*':ab,ti OR 'cryptogenic embolism stroke*':ab,ti OR 'embolic stroke*':ab,ti OR 'cardioembolic stroke*':ab,ti OR 'cardio embolic stroke*':ab,ti OR 'thrombotic stroke*':ab,ti OR 'brain infarction*':ab,ti OR 'brain isch*':ab,ti OR 'cerebral isch*':ab,ti OR 'cerebral infarction*':ab,ti OR 'cerebellar infarction*':ab,ti OR infarct*:ab,ti OR ischemia:ab,ti)) AND (('circulation'/exp OR 'basilar artery'/exp OR 'superior cerebellar artery'/exp OR 'posterior inferior cerebellar artery'/exp OR 'posterior cerebral artery'/exp OR 'anterior inferior cerebellar artery'/exp OR 'vertebral artery'/exp OR 'cerebellum'/exp OR 'occipital lobe'/exp OR 'brain stem'/exp) OR (('posterior cerebral':ab,ti OR vertebral:ab,ti OR cerebral:ab,ti OR basilar:ab,ti) AND (arteries:ab,ti OR artery:ab,ti) OR posterior:ab,ti OR fossa:ab,ti OR cerebell*:ab,ti OR occipital:ab,ti)) AND (('decompression surgery'/exp OR 'skull surgery'/exp OR 'ventriculostomy'/exp) OR ((craniotomy:ab,ti OR craniectomy:ab,ti OR hemicraniectomy:ab,ti) AND decompress*:ab,ti OR 'suboccipital craniectomy':ab,ti OR 'neurosurgical procedures':ab,ti OR ((ventriculoperitoneal:ab,ti OR 'ventriculo peritoneal':ab,ti) AND shunt*:ab,ti) OR ventriculostomy:ab,ti OR (surgical:ab,ti AND decompression*:ab,ti)))

Cochrane – 123 results

Print:

ID	Search	Hits
#1	MeSH descriptor: [Brain Infarction] explode all trees	1312
#2	MeSH descriptor: [Ischemic Stroke] explode all trees	71
#3	MeSH descriptor: [Brain Ischemia] explode all trees	3688
#4	MeSH descriptor: [Infarction] explode all trees	12383
#5	MeSH descriptor: [Ischemia] explode all trees	14276
#6	(ischemic stroke* OR ischaemic stroke* OR wake up stroke* OR cryptogenic stroke* OR cryptogenic embolism stroke* OR embolic stroke* OR cardioembolic stroke* OR cardio embolic stroke* OR thrombotic stroke* OR brain infarction* OR brain isch* OR cerebral isch* OR cerebral infarction* OR cerebellar infarction* OR infarct* OR isch*):ti,ab,kw	76333

#7 #1 OR #2 OR #3 OR #4 OR #5 OR #6 76423

#8 MeSH descriptor: [Posterior Cerebral Artery] explode all trees 9

#9 MeSH descriptor: [Vertebral Artery] explode all trees 66

#10 MeSH descriptor: [Basilar Artery] explode all trees 30

#11 MeSH descriptor: [Blood Circulation] explode all trees 7987

#12 MeSH descriptor: [Occipital Lobe] explode all trees 278

#13 MeSH descriptor: [Cerebellum] explode all trees 211

#14 ((posterior cerebral OR vertebral OR cerebral OR basilar) AND (arteries OR artery) OR posterior OR fossa OR cerebell* OR occipital):ti,ab,kw 26729

#15 #8 OR #9 OR #10 OR #11 OR #12 OR #13 OR #14 33965

#16 MeSH descriptor: [Decompression, Surgical] explode all trees 1148

#17 MeSH descriptor: [Craniotomy] explode all trees 447

#18 MeSH descriptor: [Neurosurgical Procedures] explode all trees 6202

#19 (((craniotomy OR craniectomy OR hemicraniectomy) AND decompress*) OR suboccipital craniectomy OR neurosurgical procedures OR ((ventriculoperitoneal OR ventriculo peritoneal) AND shunt*) OR (Ventriculostomy OR (surgical AND decompression*))):ti,ab,kw 2655

#20 #16 OR #17 OR #18 OR #19 8543

#21 #7 AND #15 AND #20 123

Supplementary Table S2a: The Newcastle-Ottawa Scale for risk of bias assessment of Cohort studies.

		Selection (Max ☆)				Comparability (Max ☆☆)	Outcome (Max ☆ per item)			
**No.**	**Author**	**Representativeness of the exposed cohort**	**Selection of the non-exposed cohort**	**Ascertainment of exposure**	**Demonstration that outcome of interest was not present at start of study**	**Comparability of cohorts on the basis of the design or analysis controlled for confounders**	**Assessment of outcome**	**Was follow-up long enough for outcomes to occur**	**Adequacy of follow-up of cohorts**	**Overall Quality**
1	Raco et al., 2003	☆	☆	☆	☆		☆			Poor
2	Mathew et al., 1995	☆	☆	☆	☆		☆			Poor
3	Hornig et al., 1994	☆	☆	☆	☆		☆			Poor
4	Jauss et al., 1992	☆	☆	☆	☆	☆	☆	☆	☆	Good
5	Mostofi, 2013	☆	☆	☆	☆		☆	☆		Good
6	Koh et al., 1999	☆	☆	☆	☆		☆	☆	☆	Good
7	Taneda et al., 1982	☆	☆	☆	☆		☆			Poor
8	Taylor et al., 2020	☆	☆	☆	☆	☆	☆			Good

Supplementary Table S2b: The Joanna Briggs Institute (JBI) Critical Appraisal Checklist for analytical case-series.

No.	Author	Were there clear criteria for inclusion in the case series?	Was the condition measured in a standard, reliable way for all participants included in the case series?	Were valid methods used for identification of the condition for all participants included in the case series?	Did the case series have consecutive inclusion of participants?	Did the case series have complete inclusion of participants?	Was there clear reporting of the demographics of the participants in the study?	Was there clear reporting of clinical information of the participants?	Were the outcomes or follow up results of cases clearly reported?	Was there clear reporting of the presenting site(s)/clinic(s) demographic information?	Was statistical analysis appropriate?
1	Tsitsopoulos et al., 2011	Yes	Yes	Yes	Yes	Yes	Yes	Yes	Yes	No	Yes
2	Baldauf et al., 2006	Yes	Yes	Yes	Yes	Yes	Yes	Yes	Yes	No	Yes
3	Koh et al., 2000	Yes	Yes	Yes	Yes	Yes	Yes	Yes	Yes	No	Yes
4	Pfefferkorn T et al, 2009	Yes	Yes	Yes	No	Yes	Yes	Yes	Yes	Yes	Yes
5	Jüttler et al., 2009	Yes	Yes	Yes	Yes	Yes	Yes	Yes	Yes	Yes	Yes
6	Lee et al., 2020	Yes	Yes	Yes	No	Yes	Yes	Yes	Yes	Yes	No
7	Tsitsopoulos et al., 2011	Yes	Yes	Yes	No	Yes	Yes	Yes	Yes	Yes	Yes
8	Chen et al., 1992	Yes	Yes	Yes	No	Yes	Yes	Yes	Yes	Yes	No
9	Moussa et al., 2013	Yes	No	No	No	No	Yes	Yes	Yes	Yes	No
10	Tartara et al., 2018	Yes	Yes	No	No	No	Yes	Yes	Yes	Yes	Yes
12	Kudo et al., 2007	Yes	No	No	No	No	Yes	Yes	Yes	Yes	Yes
13	Krieger et al., 1993	Yes	Yes	Yes	No	No	No	No	Yes	No	No
15	Yoshio et al., 2019	Yes	Yes	Yes	Yes	Yes	Yes	Yes	Yes	Yes	No
16	Lindeskoga et al., 2018	Yes	Yes	Yes	No	Yes	Yes	Yes	Yes	Yes	Yes
17	Mattar et al., 2021	Yes	Yes	Yes	No	Yes	Yes	Yes	Yes	Yes	Yes
18	Hernández-Durán et al., 2020	Yes	Yes	Yes	No	Yes	Yes	Yes	Yes	Yes	Yes
19	Goedemans et al., 2017	Yes	Yes	Yes	No	Yes	Yes	Yes	Yes	Yes	Yes
20	Yoshimura, et al., 2007	Yes	Yes	Yes	No	Yes	Yes	Yes	Yes	Yes	No
21	Lee et al., 2014	Yes	Yes	Yes	No	No	Yes	Yes	No	Yes	Yes
22	Mangubat et al., 2009	Yes	Yes	Yes	Yes	Yes	Yes	Yes	Yes	Yes	Yes
23	Vindigni et al., 2010	Yes	Yes	Yes	No	No	Yes	Yes	Yes	Yes	No
24	Bertalanffy et al., 1992	Yes	Yes	Yes	Yes	Yes	Yes	Yes	Yes	Yes	No

Supplementary Table S2c: The Joanna Briggs Institute (JBI) Critical Appraisal Checklist for analytical case-control studies.

No.	Author	Were the groups comparable other than the presence of disease in cases or the absence of disease in controls?	Were cases and controls matched appropriately?	Were the same criteria used for identification of cases and controls?	Was exposure measured in a standard, valid and reliable way?	Was exposure measured in the same way for cases and controls?	Were confounding factors identified?	Were strategies to deal with confounding factors stated?	Were outcomes assessed in a standard, valid and reliable way for cases and controls?	Was the exposure period of interest long enough to be meaningful?	Was appropriate statistical analysis used?
1	Kim et al., 2016	Yes	Yes	Yes	Yes	Yes	Yes	Yes	Yes	Yes	Yes
